# Optimizing 2D in vitro differentiation conditions for C2C12 murine myoblasts on gelatin hydrogel

**DOI:** 10.1007/s10974-025-09711-0

**Published:** 2025-10-09

**Authors:** Veronica Sian, Per Harald Jonson, Anna Vainio, Helena Luque, Swethaa Natraj Gayathri, Peter Hackman, Bjarne Udd, Marco Savarese, Jaakko Sarparanta

**Affiliations:** 1 Folkhälsan Research Center, Helsinki, Finland; 2 University of Helsinki, Helsinki, Finland; 3 Neuromuscular Research Centre, Tampere University and University Hospital, Tampere, Finland

**Keywords:** C2C12 cells, Differentiation, Myoblasts, Myotubes, Twitching, Insulin

## Abstract

Optimizing in vitro differentiation protocols for skeletal muscle cells is essential for producing mature, functional myotubes suitable for disease modeling and therapeutic screening. While C2C12 murine myoblasts are a widely used model, achieving consistent and advanced differentiation remains challenging. In this study, we systematically evaluated conditions that improve myotube formation and maturation in a 2D culture system using ultra-compliant gelatin hydrogels. We compared standard and commercial differentiation media and identified that supplementation of DMEM with 2% horse serum and 10% Opti-MEM (DMO) supported robust myotube formation, with thin and aligned fibers. Insulin supplementation significantly increased expression of myosin heavy chain (MyHC) and calsequestrin, while pyruvate provided additional benefit by further enhancing myotube maturation. Media change frequency was also critical: daily replacement was necessary to maintain optimal differentiation, although the addition of insulin and pyruvate partly mitigated the effects of less frequent changes. Application of electrical pulse stimulation (EPS) improved sarcomeric α-actinin organization without significantly altering MyHC isoform expression. RNA sequencing confirmed transcriptional reprogramming consistent with myogenic progression, including early upregulation of key muscle-specific genes. Our findings present a cost-effective, reproducible protocol that supports advanced C2C12 differentiation in a scalable 2D system, offering practical guidance for generating mature, functional myotubes in vitro for both basic and translational muscle research.

**Supplementary Information:**

The online version contains supplementary material available at 10.1007/s10974-025-09711-0.

## Introduction

Skeletal muscle is one of the largest and most dynamic organs in the body, playing a fundamental role not only in locomotion but also in whole-body metabolic regulation, glucose homeostasis, and thermogenesis (Baskin et al. 2015; Collins and Partridge 2005; Egan and Zierath 2013; Greggio et al. 2017; Rowland et al. 2015). The intricate process of myogenesis, which governs both muscle development and regeneration following injury, involves the activation of muscle progenitor cells known as satellite cells (Almeida et al. 2016; Karalaki et al. 2009; Yin et al. 2013). Upon activation, these cells proliferate as mononuclear myoblasts before undergoing differentiation and fusion to form multinucleated myotubes, which subsequently mature into functional myofibers (Wong et al. 2020; Yin et al. 2013).

Beyond its contractile function, skeletal muscle is a highly heterogeneous tissue composed of multiple cell types, including motor neurons, endothelial cells, fibroblasts, pericytes, resident immune cells, and smooth muscle cells that contribute to its structural integrity, vascularization, and response to physiological stimuli (Giordani et al. 2019; Mukund and Subramaniam 2020). This cellular complexity presents significant challenges in isolating the specific role of muscle cells in vivo, particularly when investigating intramyocellular signalling, metabolic adaptations, and pathological conditions. As a result, researchers have increasingly turned to in vitro models to study skeletal muscle biology under controlled conditions (Cao and Warren 2025; Zschüntzsch et al. 2022).

Among the various in vitro models available (Cao and Warren 2025), the murine C2C12 myoblast cell line has long been established as one of the most widely used and well-characterized systems for investigating muscle development, differentiation, and function (Falcieri et al. 2004). C2C12 cells proliferate as mononucleated myoblasts and differentiate into multinucleated myotubes when cultured in appropriate differentiation media (Denes et al. 2019). This model provides a reproducible and scalable platform for studying key aspects of myogenesis, gene expression regulation, metabolic responses, and muscle-specific signalling pathways (Abdelmoez et al. 2020). Furthermore, C2C12 cells have been extensively utilized for drug screening, disease modelling, and the investigation of muscle atrophy, hypertrophy, and regeneration (Ikeda et al. 2017; Li et al. 2023; Zschüntzsch et al. 2022).

Despite the widespread use of this model, standardized and optimized protocols for 2D culturing remain variable across laboratories, particularly in terms of media composition, supplement concentrations, and differentiation timelines. These parameters, together with cell density and passage number, can significantly impact the efficiency of myotube formation, gene expression dynamics, and the overall reliability of the model, especially when used for transcriptomic or pharmacological studies.

In this study, we systematically optimized the 2D in vitro culturing conditions for C2C12 cells by evaluating the impact of different culture media and supplement combinations on the differentiation process. We performed RNA sequencing (RNA-seq) analysis of cells at multiple time points, covering the transcriptional dynamics from proliferating myoblasts to differentiated myotubes. This work aims to provide a practical and reproducible framework for researchers utilizing the C2C12 model, supporting improved standardization across laboratories and enhancing the utility of this system in muscle biology research.

## Materials and methods

### C2C12 cell culture

C2C12 murine myoblasts were purchased from ATCC (C2C12 - CRL-1772). Cells were cultured in Dulbecco’s modified Eagle medium (DMEM, Gibco), without phenol-red and pyruvate, supplemented with 20% fetal bovine serum (SERANA S-FBS-SA-015), 1× GlutaMAX (Gibco) and 1× penicillin/streptomycin (Gibco) in incubator at 37 °C under humidified atmosphere of 5% CO2. Cells were subcultured when they reached 50% of confluence.

Ultra-compliant gelatin hydrogels were prepared essentially as described (Jensen et al. 2020). Briefly, 5% (wt/vol) type A porcine gelatin (Sigma-Aldrich G2625) and 20 U/mL microbial tranglutaminase (Bindly TI, BDF Natural Ingredients, Girona, Spain) were prepared in sterile PBS and filtered through 0.45-µm filters. The two solutions were combined 1:1, cast to cell culture plasticware ~ 130 µl/cm^2^, solidified in the fridge 15 min, and crosslinked in 37 °C for 4 h. Hydrogels were then washed with PBS three times before plating the cells. For differentiation, C2C12 myoblasts between passage 4 and passage 8 were grown on gelatin hydrogels to confluency and differentiated to myotubes up to 21 days.

### Media test for differentiation 

Different media compositions have been used to differentiate C2C12 cells (see Supplementary Table S1). The following media and supplements were used: Dulbecco’s modified Eagle medium (DMEM, Gibco), horse serum (Gibco 26050088), pen-strep (Gibco 15140-122), L-glutamine (Euroclone ECB3000D), Opti-MEM (Gibco 31985-047), Insulin-Transferrin-Selenium (ITS-G; Gibco 41400-045), pyruvate (Gibco 11360-070), Skeletal Muscle Cell Growth Medium (PromoCell C-23060), Skeletal Muscle Differentiation Medium (PromoCell C-23061). Cells were analysed at 1, 2, and 3 weeks following the initiation of differentiation.

### Electrical pulse stimulation

Where noted, the C2C12 cells were electrically stimulated during the last two days of differentiation using a C-Pace EM system (Ionoptix, MA, USA). The default pulse settings for 12-well plates (Falcon, 353043) were 4.8 V, 6 ms pulses at 2 Hz, whereas tetanic stimulation was performed at 4.8 V, using 2 ms long pulses at 30 Hz for 5 s followed by a 25 s relaxation period.

### SDS-PAGE and western blotting

For western blotting analyses, protein samples were separated in Criterion TGX gels 4–15% (Bio-Rad, Hercules, CA, USA) and transferred on nitrocellulose membranes with the Trans-Blot Turbo system (Bio-Rad). Total protein was stained with the Revert 520 Total Protein Stain (LI-COR Biosciences, Lincoln, NE, USA) and scanned with an Odyssey M scanner (LI-COR). Blots were stained with the following primary antibodies: anti-myosin heavy chain MF20 (DSHB), anti-Calsequestrin 1/2 (Abcam, ab3516), anti-Troponin T1 (HPA058448, Sigma-Aldrich; RRID: AB_2683720), anti-troponin T3 (clone T1/61, Novocastra), anti- fast myosin heavy chain F59 (DSHB), anti- slow myosin heavy chain BA-D5 (DSHB), anti-embryonic myosin F1.652-s (DSHB). Detection was performed with fluorescent secondary antibodies using an Odyssey M scanner. Image analysis was done in Fiji (Schindelin et al. 2012).

### Vertical agarose gel electrophoresis (VAGE)

Differentiated C2C12 cells and mouse control tissue (left ventricle and soleus) were lysed in 40 µl/mg sample buffer (8 M urea, 2 M thiourea, 3% SDS, 75 mM fresh DTT, 0.05 M tris HCl pH 6.8) using a pestle in a water bath at 60 °C for 30 s before addition of 4.8 µl 50% glycerol with protease inhibitors (1x HALT) and a further 30 s in the water bath. The samples were then centrifuged for 5 min at 13,000 rpm (15,871 g) and the supernatant aliquoted before being flash frozen and stored at −80 °C. Before gel run the samples were melted at 60 °C and recentrifuged. Expression of titin protein was examined using 1% agarose gels (Warren et al. 2003) and staining with SimplyBlue SafeStain (Invitrogen, 465044).

### Cell morphology

Cell morphology was observed under the brightfield phase-contrast microscope (Nikon Eclipse Ts2). The images were acquired with the 4× magnification.

### Immunofluorescence

C2C12 grown on gelatin hydrogel cells were fixed with 4% PFA, permeabilized with 0.02% Triton X-100 for 10 min at RT. Samples were blocked with 5% BSA for 1 h at RT, and then incubated with primary antibody anti- Sarcomeric alpha-actinin (EA-53) overnight at 4 °C. Secondary antibody was incubated for 1 h at RT protected from light. Hoechst was added for 5 min at RT. For mounting with PVA mounting media (10% wt/vol polyvinyl alcohol, 25% vol/vol glycerol, 100 mM Tris-HCl pH 8.5), the hydrogels were equilibrated in mounting media : PBS (50:50) 1–2 h RT, in mounting media overnight at + 8 °C, and finally in mounting media 1–2 h RT. After removing the excess mounting media, a #1.5 coverslip was applied, and the samples were dried in room temperature. Zeiss Axio Imager M2 using 40× NA 1.3 oil immersion objective (Carl Zeiss AG, Oberkochen, Germany) was used for the image acquisitions.

### Transcriptomic analysis

C2C12 grown on hydrogel were differentiated in differentiation media (DMO) and collected after 3, 7, and 16 days of differentiation using Collagenase type I 1 mg/mL (Gibco). A total of 18 samples were processed for transcriptome sequencing (see Supplementary Table S2). Total RNA was extracted with Trizol (Invitrogen). RNA concentration and purity was measured using NanoDrop 2000 (Thermo Fisher Scientific, Wilmington, DE). RNA integrity was assessed using the RNA Nano 6000 Assay Kit of the Agilent Bioanalyzer 2100 system (Agilent Technologies, CA, USA). mRNA was purified from total RNA using oligo-dT-attached magnetic beads. Sequencing libraries were generated using NEBNext UltraTM RNA Library Prep Kit for Illumina (New England Biolabs, USA) following manufacturer’s recommendations.

Library was sequenced in Illumina NovaSeq6000. Hisat2 tools software was used to map with reference genome (Mus_musculus GRCm39). About 20 million reads per sample were generated.

Differential expression analysis was performed using DESeq2 (Love et al. 2014). P-values were adjusted using the Benjamini and Hochberg’s approach for controlling the false discovery rate. Genes with an adjusted p-value < 0.01 were assigned as differentially expressed. Gene ontology (GO) analysis of differentially expressed genes (DEGs) was performed in three categories: biological process (BP), cellular component (CC), and molecular function (MF). One sample was excluded from the analysis as it failed data quality control. The analysis was performed using BMKCloud (www.biocloud.net). Volcano plots were generated using Python.

### Differential splicing analysis methods

Differential splicing analysis was performed using Modeling Alternative Junction Inclusion Quantification (MAJIQ) v2 (Green et al. 2018). MAJIQ Quantifier’s *delta percentage spliced in* (ΔPSI) function was used to quantify changes in local splice variations (LSVs) between sample groups. Default settings were applied, considering splice junctions as differentially spliced if they exhibited a change of |ΔPSI| ≥ 0.2 (20%) with a confidence threshold of ≥ 0.05. The analysis pipeline required that splice junctions be present in at least 80% of samples within each comparison group, using the GRCm39 GENCODE Release M33 annotation files as reference. Gene Ontology enrichment analysis was performed using g: Profiler in R (Kolberg et al. 2023; Reimand et al. 2007). Multiple testing correction was applied using the Benjamini–Hochberg false discovery rate (FDR) method. To address redundancy in the enrichment results, GO terms were grouped based on semantic similarity using the rrvgo package in R (Sayols 2023). The grouping was performed with a similarity threshold of 0.8, and scores were based on –log10(p-value). Treemaps summarizing the grouped terms were generated with treemapPlot.

### Statistical analysis

A one-way ANOVA with Tukey’s multiple comparisons was performed to determine significant relationships between different conditions and DMO in western blot analyses. DMO was used as reference control (= 1). Statistical significance for these experiments was set a priori at *p* < 0.5. Data are represented as mean ± SD.

## Results

**Fig. 1:**
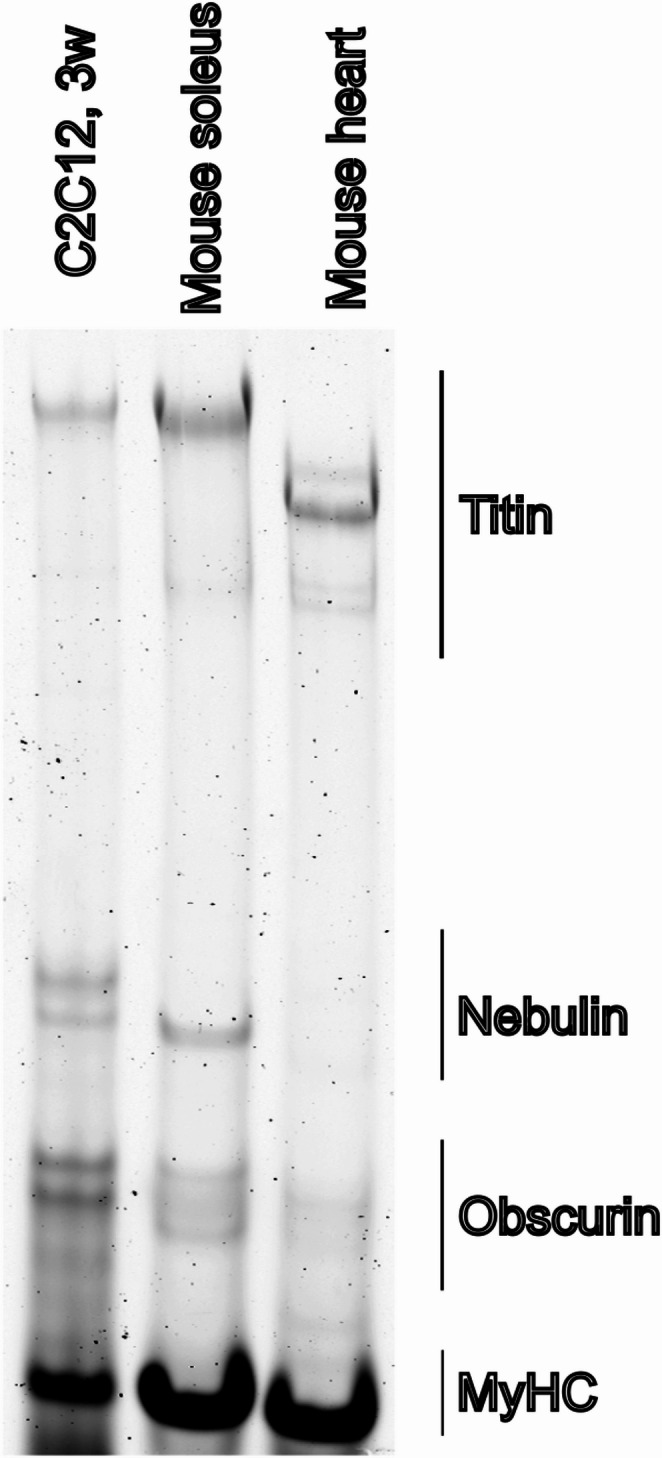
Titin protein expression in C2C12 myotubes. Titin protein was detected in C2C12 differentiated on hydrogel for 3 weeks (C2C12, 3w) using vertical agarose gel electrophoresis (VAGE). Titin full length was detected in C2C12 cells, and mouse muscle soleus and mouse heart were run as controls

The standard differentiation medium (DM) widely used for C2C12 differentiation is DMEM supplemented with 2% heat-inactivated horse serum (HS). However, the HS batches are known to differ in the ability to support differentiation, and our experience is that identifying satisfactorily performing batches is challenging. We have observed that DM further supplemented with 10% OPTI-MEM (DMO) consistently shows reliable induction of myogenic differentiation, and we have adopted DMO as the standard C2C12 differentiation medium in our laboratory. We noticed that after 3 weeks of differentiation on gelatin hydrogels C2C12 myotubes expressed full length Titin (Fig. 1). However, to better understand the factors influencing differentiation efficiency, we sought to investigate whether additional components—such as sodium pyruvate or insulin—or variables such as the frequency of media changes, or other commercial media could further enhance or modulate the differentiation outcome.

### Comparison of differentiation medium and promocell medium

**Fig. 2:**
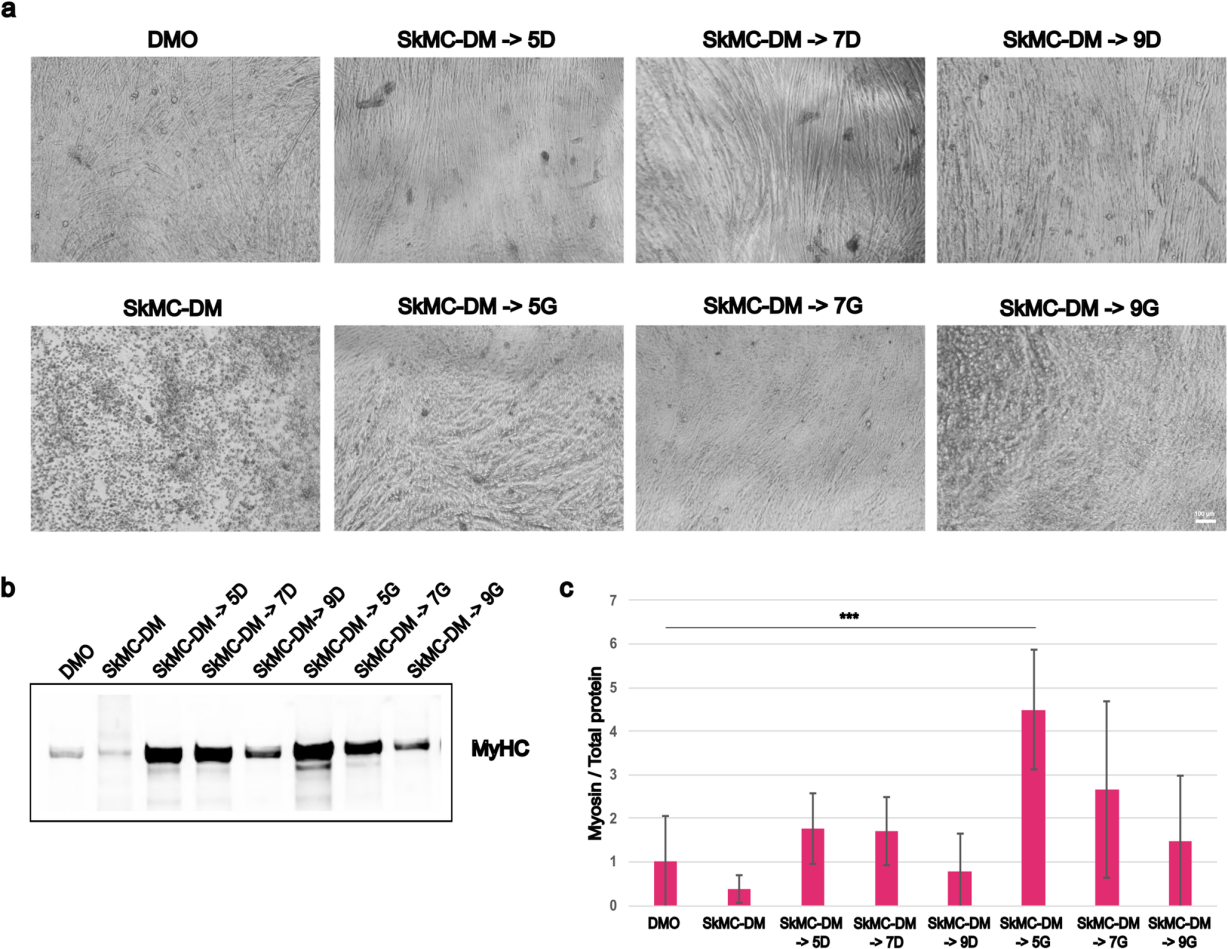
Optimization of the most effective conditions for PromoCell differentiation medium. Comparison of differentiation medium (DMO) and PromoCell differentiation medium (SkMC-DM), and determination of optimal time to switch to DMO or growth medium for C2C12 cells. Cells were differentiated for 3 weeks on hydrogels. (**a**) Light microscopy showing cell morphology for the various culture strategies after 21 days. Daily changes of DMO or SkMC-DM for the whole time or changed from SkMC-DM to DMO after five, seven or nine days (SkMC-DM ->5D, SkMC-DM ->7D, SkMC-DM ->9D) or to PromoCell growth medium after five, seven or nine days (SkMC-DM ->5G, SkMC-DM ->7G, SkMC-DM ->9G). (**b**) Representative western blot for myosin heavy chain (MyHC). In (**c**) the relative level of myosin to total protein for the same culture conditions (three asterisks indicate *p* < 0.001). DMO was used as control (= 1). Graph shows mean ± SD of six wells from two replicate experiments (for the stain see Supplementary Figure S5)

We first compared DMO with a commercially available skeletal muscle differentiation medium (SkMC-DM) from PromoCell. This initial comparison aimed to determine how DMO formulation performs relative to a standardized commercial option. We had previously observed that C2C12 initially differentiate efficiently in PromoCell SkMC-DM, but deteriorate if kept in this medium longer. Indeed, the manufacturer’s protocol for human primary myoblasts advises changing the medium to Skeletal Muscle Cell Growth Medium (SkMC-GM) after a few days. To determine a protocol that works for C2C12 cells on hydrogel, we tested differentiation in DMO, PromoCell SkMC-DM alone, and conditions where cells were initially differentiated in PromoCell SkMC-DM and then switched either to PromoCell SkMC-GM (SkMC-DM->G) or DMO (SkMC-DM->D) after 5, 7, or 9 days, with daily media changes. Cell morphology was documented at 1, 2 (see Supplementary Fig. 1), and 3 weeks (Fig. 2a) following the initiation of differentiation. Phase-contrast imaging confirmed that after initial myotube formation, cells did not thrive in PromoCell SkMC-DM. However, switching to SkMC-GM (SkMC-DM->G) or DMO (SkMC-DM->D) after 5 or 7 days of differentiation appeared to mitigate this effect. Expression of myosin heavy chain (MyHC), a marker of myogenic differentiation, was assessed at the 3-week time point to evaluate the extent of terminal differentiation. In terms of myosin content, the clearly most effective condition to promote myotube formation was a switch to SkMC-GM after 5 days of differentiation (Fig. 2b-c). Interestingly, while spontaneous twitching was observed after 3 weeks with both SkMC-GM and DMO, the two media resulted in different myotube morphology: SkMC-DM followed by DMO (SkMC-DM->D) produced thinner aligned myotubes—similarly to DMO alone—whereas switching to SkMC-GM (SkMC-DM->G) gave produced thicker and less organized myotubes.

### Effect of media composition to myogenic differentiation

**Fig. 3:**
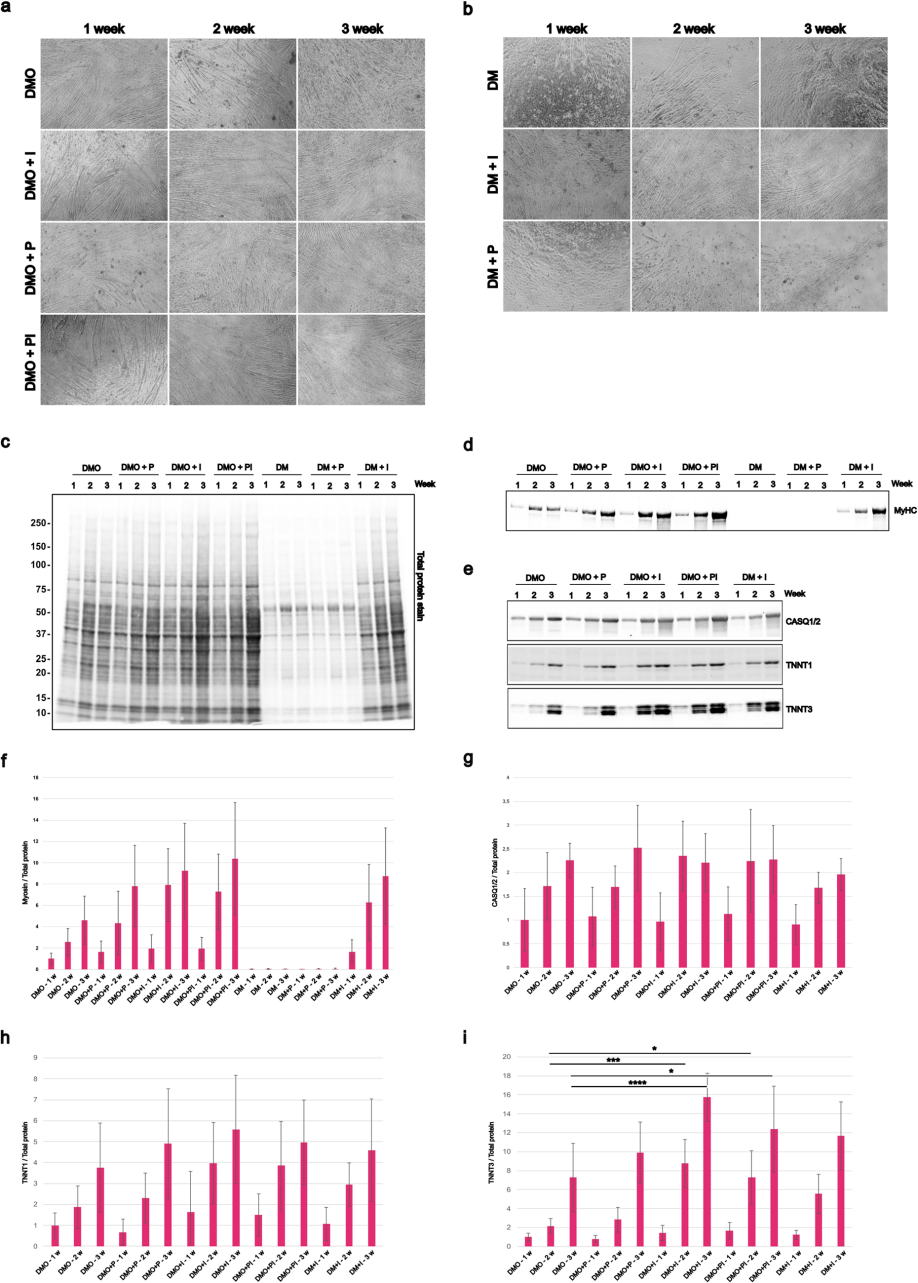
Effects of different media composition. (**a**) Phenotypic effects of supplementing standard differentiation medium (DMO). Light microscopy shows myotube formation in C2C12 cells cultured in DMO, DMO + I (insulin, transferrin and selenium), DMO + P (pyruvate), or DMO + PI after 1, 2, and 3 weeks. Scale bar 100 μm. (**b**) Morphological effects of Opti-MEM omission in differentiation medium (DM). Light microscopy shows myotube development in C2C12 cells cultured in DM, DM + I or DM + P after 1, 2, and 3 weeks. Scale bar 100 μm. (**c**) Total protein stain for MyHC after 1, 2, and 3 weeks of differentiation in different media composition. C2C12 were cultured in DMO and DM supplemented with Insulin-Transferrin-Selenium (DM + I, DMO + I), and with pyruvate (DMO + P, DM + P), and DMO supplemented with both Insulin-Transferrin-Selenium and pyruvate (DMO + PI). Representative western blot for (**d**) myosin heavy chain (MyHC), (**e**) calsequestrin 1/2 (CASQ1/2), Troponin T1 (TNNT1), and Troponin T3 (TNNT3). Quantification of **f**) MyHC **g**) CASQ1/2, **h**) TNNT1, **i**) TNNT3 on total protein stain (for the stain see Supplementary Figure S5). Graph shows mean ± SD of nine wells from two replicate experiments for myosin heavy chain and calsequestrin1/2, and six wells from two replicate experiments for Troponin T1 and T3. One asterisk indicates *p* < 0.5, three asterisks indicate *p* < 0.001, four asterisks indicate *p* < 0.0001

We then investigated the contribution of individual media components by modifying the differentiation medium formulation. These experiments aimed to identify whether specific supplements could enhance differentiation or compensate for the removal of key components. Insulin is known to promote myotube formation (Conejo et al. 2001; Florini et al. 1996), and we assume that the good performance of DMO compared to DM depends on insulin present in Opti-MEM. Hence, we tested the effect of supplementing standard DM or DMO with Insulin-Transferrin-Selenium (DM + I, DMO + I). Some online sources advise against including pyruvate in differentiation medium (Jiang et al. 2023), and hence we use base DMEM without pyruvate, but we reasoned that an additional carbon source could be beneficial for the high metabolic demands of the myotubes. To test this, DM and DMO were supplemented with 1 mM sodium pyruvate (DM + P, DMO + P). Additionally, DMO was supplemented with both insulin-transferrin-selenium (I) and pyruvate (DMO + PI). Differentiation outcomes were assessed through morphological analyses (Fig. 3a and b) and quantification of myosin heavy chain (MyHC), calsequestrin 1/2 (CASQ1/2), Troponin T1 (TNNT1) and T3 (TNNT3) expression at 1, 2, and 3 weeks after starting the differentiation (Fig. 3c-g).

Phase-contrast imaging for DMO and its modified conditions revealed no notable differences in myotube formation (Fig. 3a). The total cellular biomass progressively increased with the duration of differentiation, reflecting the accumulation of mature, multinucleated myotubes over time (Fig. 3c). Total MyHC expression progressively increased over time, with detectable levels rising from week 1 through week 3 (Fig. 3d and f). Both insulin-transferrin-selenium and pyruvate supplementation, together or in combination, enhanced MyHC expression compared to DMO alone, with insulin-transferrin-selenium producing a more pronounced effect.

In contrast, omission of Opti-MEM (DM) impaired myotube development (Fig. 3b) and MyHC production (Fig. 3d and f), indicating that the utilized horse serum lot does not support differentiation effectively. This was notably rescued by the addition of insulin-transferrin-selenium (DM + I), whereas supplementation with pyruvate (DM + P) did not result in any visible improvement in myotube morphology and myosin production.

Due to the low expression of total myosin, we excluded DM and DM + P from western blot analyses of CASQ1/2, TNNT1, and TNNT3 at week 1, 2, and 3 of differentiation (Fig. 3e). At week 3, DMO significantly increased calsequestrin levels, and also insulin-transferrin-selenium and pyruvate, both individually and combined, further elevated its expression (Fig. 3g). The same effects can be observed in the TNNT1 and TNNT3 protein expression (Fig. 3e and h-i). Notably, both DMO + I and DMO + PI significantly increased TNNT3 expression after 2 and 3 weeks of differentiation (Fig. 3i). Supplementation with insulin-transferrin-selenium seemed also to prevent the embryonic myosin downregulation at week 2 (*p* < 0.05) and 3 of differentiation compared to the DMO control condition, as observed by western blot analysis (Supplementary Figure S2).

### Effect of media change frequency on C2C12 differentiation

**Fig. 4:**
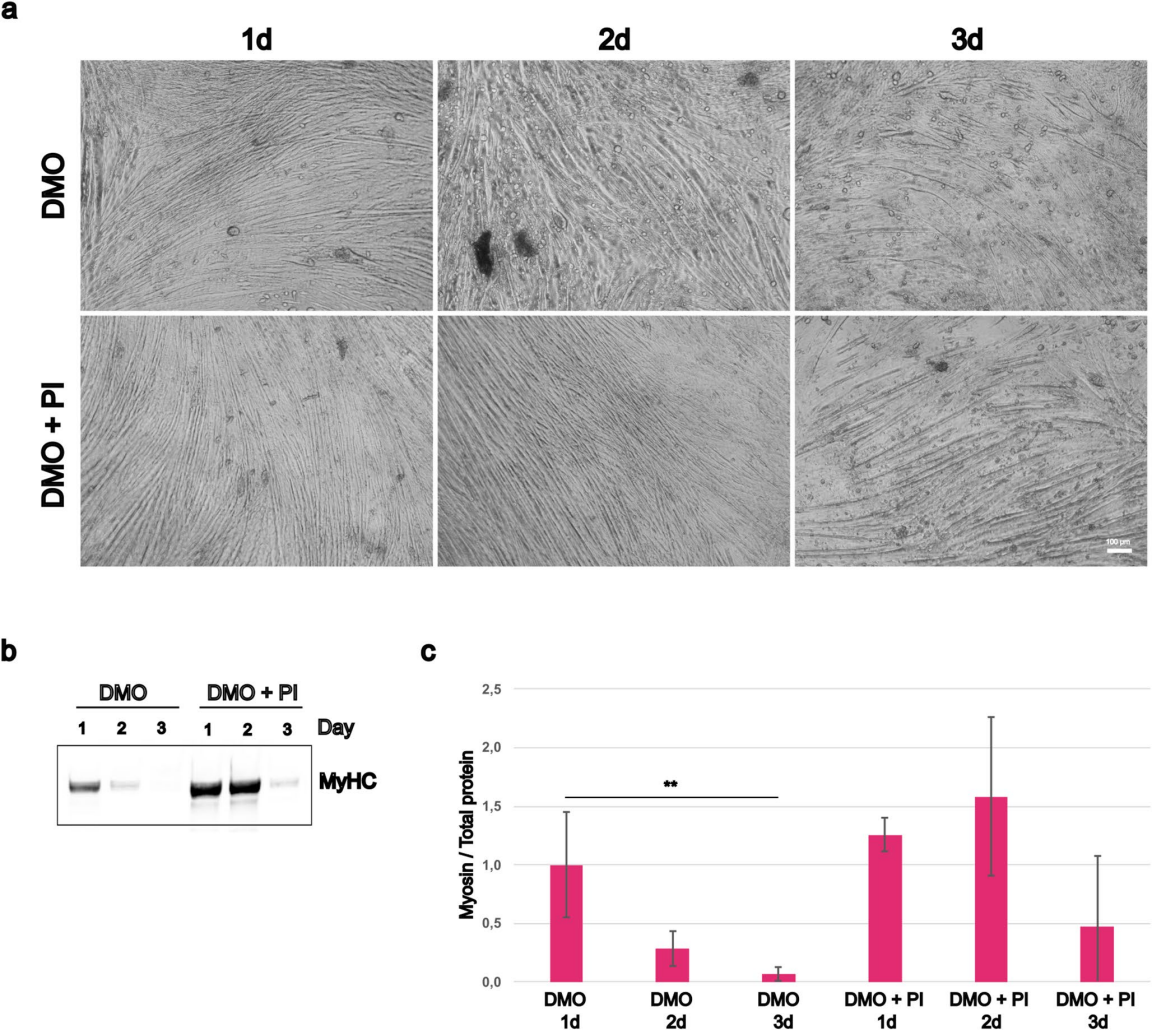
Impact on media change schedule on C2C12 differentiation. (**a**) Cells were cultured in DMO or DMO + PI for three weeks with media changes daily (1d), every second day (2d) or every third day (3d). In (**b**) representative western blot of myosin heavy chain (MyHC) and in (**c**) quantification of total myosin versus total protein (two asterisks indicate *p* < 0.01). DMO 1 d was used as control (= 1). Graph shows mean ± SD of six wells from two replicate experiments (for the stain see Supplementary Figure S5)

C2C12 culture protocols typically advise to change differentiation media daily, although variations exist. As less frequent media changes would reduce both the workload and cost of experiments, we evaluated the impact of media change frequency on C2C12 differentiation in the hydrogel system. Cells differentiated in DMO and DMO + PI were subjected to different schedules, with media changes performed daily, every other day, or every three days. Phase-contrast imaging revealed that daily media changes provided the most favourable condition for myotube formation and maturation (Fig. 4a). This observation was supported by western blot analysis of MyHC expression, which decreased significantly with reduced media change frequency—particularly in the DMO condition when media was changed every three days (*p* < 0.01). Interestingly, the inclusion of insulin and pyruvate (DMO + PI) helped preserve myosin levels under less frequent media change schedules (Fig. 4b-c).

### Effects of electrical pulse stimulation (EPS) on C2C12 differentiation

**Fig. 5:**
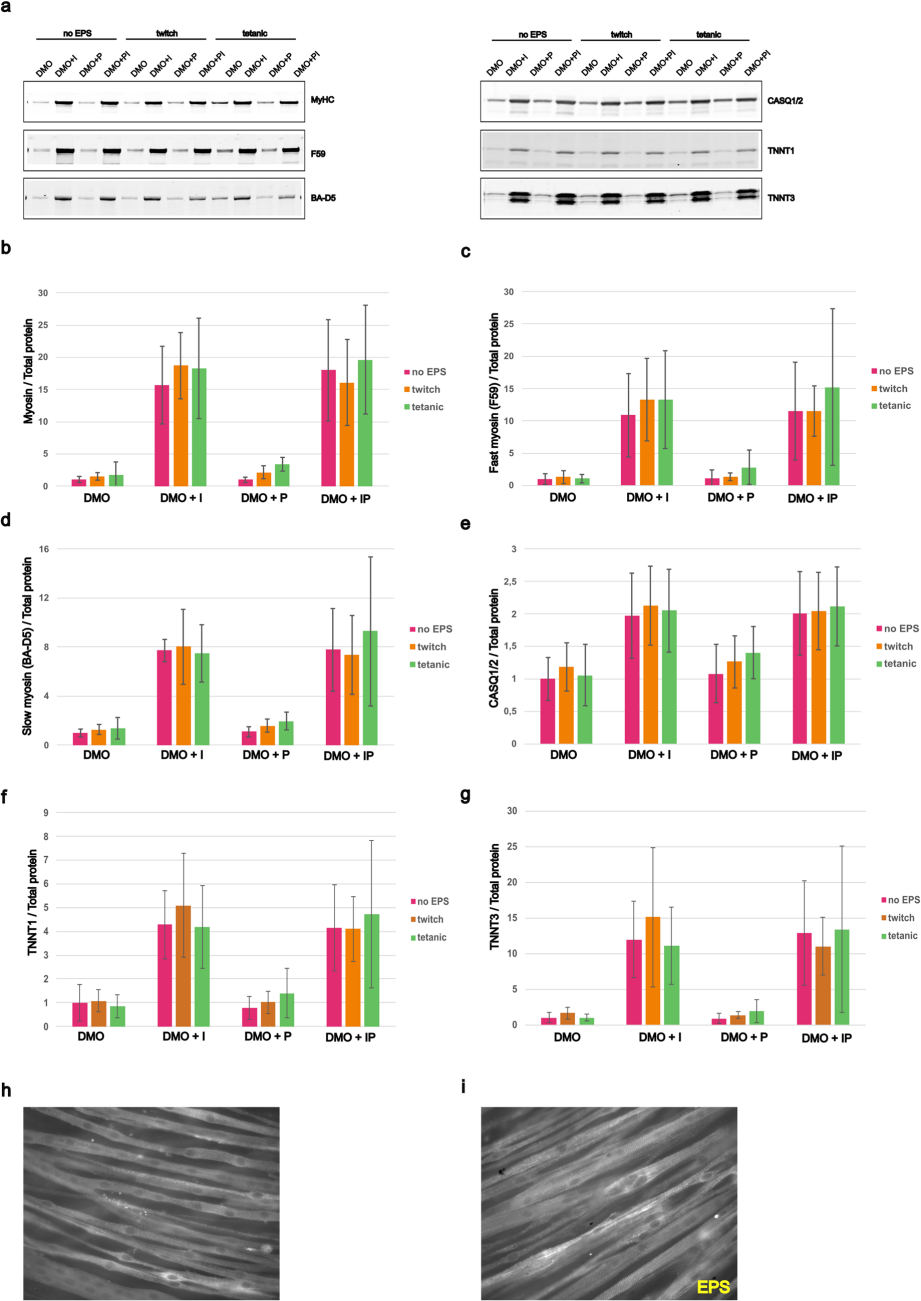
The effects of electric pulse stimulation (EPS) on C2C12 differentiation. C2C12 cells were cultured in DMO, DMO + P, DMO + I, DMO + PI for 3 weeks followed by 2 days of pulse stimulation. (**a**) Representative western blots for myosin heavy chain, fast-myosin F59, slow-myosin BA-D5, calsequestrin 1/2, troponin T1 and troponin T3. Protein quantifications on the total stain for (**b**) myosin heavy chain, (**c**) fast-myosin F59, (**d**) slow-myosin BA-D5, (**e**) calsequestrin 1/2, (**f**) troponin T1, (**g**) troponin T3. Graph shows mean ± SD of six wells from two replicate experiments. Immunofluorescence for the Z-disc marker α-actinin-2 without (**h**) and with (**i**) stimulation after 14 days of differentiation

We evaluated also the effects of electric pulse stimulation (EPS) on C2C12 differentiation under different media conditions. After three weeks of differentiation in DMO, DMO + I, DMO + P, DMO + PI, cells were subjected to EPS for two consecutive days using either a twitch or a tetanic stimulation protocol. Protein expression of total MyHC, fast MyHC, slow MyHC, CASQ1/2, TNNT1 an TNNT3 was assessed by western blot (Fig. 5a). Protein quantification revealed that, across all media conditions and stimulation types, no significant differences in these protein levels were observed between stimulated and non-stimulated groups (Fig. 5b-g). EPS could nevertheless have benefits not reflected in myosin content. Electrically stimulated myotubes showed clear contractile activity upon stimulation, indicating the presence of functional sarcomeres and, indeed, immunofluorescence analyses for sarcomeric α-actinin at day 14 of differentiation, in absence (Fig. 5h) or presence (Fig. 5i) of EPS, showed that the electric stimulation enhances the formation and organization of mature myotubes, evidenced by more defined sarcomeric structures.

### Transcriptomic analysis

**Fig. 6:**
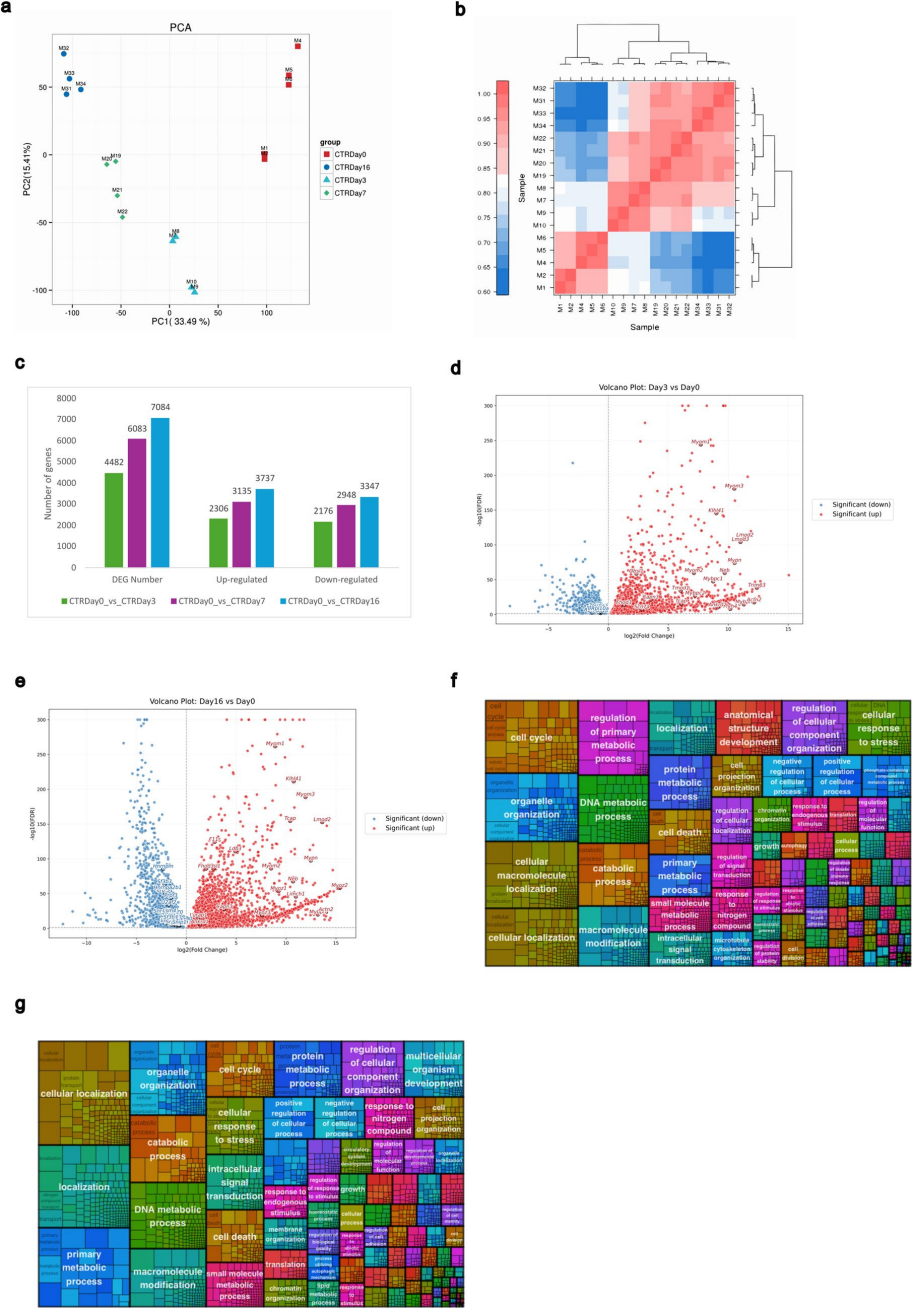
RNAseq of C2C12 cells at 3, 7 and 16 days of differentiation. **a**) Principal component analysis (PCA) shows clustering by length of differentiation and a gradual switch from day 0 to day 16 of differentiation, which is supported by clustering in **b**). **c**) The number of genes found to be statistically significantly changed in the various comparisons. **d**) Volcano plots representing differentially expressed genes at day 3 and **e**) at day 16 associated with the identified GO terms. **f**) Treemap charts illustrate significant GO-BP terms associated with differential splicing events at day 3 and **g**) day 16 of differentiation. Each rectangular shape represents individual terms, and their sizes vary according to the number of genes present in them. All the rectangles have a unique color signifying the distinct BP terms

To investigate transcriptional changes associated with C2C12 myogenic differentiation, RNA sequencing (RNA-seq) was performed on cells collected at four key time points: day 0 (undifferentiated myoblasts), and after 3, 7, and 16 days of differentiation (Supplementary Table S2). A two-dimensional Principal Component Analysis (PCA) revealed distinct clustering of samples based on differentiation stage (Fig. 6a). Specifically, samples at day 0 and day 16 showed the greatest separation along Principal Component 1 (PC1), representing the most significant source of transcriptional variance. In contrast, samples at days 3 and 7 were distinguished primarily along Principal Component 2 (PC2), suggesting a transitional gene expression profile during early and mid-differentiation stages.

The sample-to-sample correlation heatmap based on global gene expression profiles illustrates the reproducibility and stage-specific clustering of biological replicates throughout C2C12 differentiation (Fig. 6b). Day 0 samples (M1–M6) clustered tightly, reflecting their shared proliferative myoblast profile. Intermediate samples (day 3 and 7; M7–M22) showed moderate correlation, indicative of a transitional gene expression state. In contrast, day 16 samples (M31–M34) formed a separate cluster with reduced similarity to early stages, consistent with terminal differentiation into myotubes.

For differential expression analysis, pairwise comparisons were conducted using day 0 as the reference. Comparisons were made with day 3, day 7, and day 16. These analyses identified: 4,482 differentially expressed genes (DEGs) between day 0 and day 3 (group 1), including 2,306 upregulated and 2,176 downregulated genes; 6,083 DEGs between day 0 and day 7 (group 2), with 3,135 upregulated and 2,948 downregulated genes, 7,084 DEGs between day 0 and day 16 (group 3), comprising 3,737 upregulated and 3,347 downregulated genes (Fig. 6c).

To characterize the functional significance of the observed transcriptional changes, Gene Ontology (GO) enrichment analysis was performed on the upregulated and downregulated gene sets from groups 1 and 3, representing early and late stages of differentiation, respectively.

In group 1 (day 0 vs. day 3), upregulated genes were enriched in BPs associated with skeletal muscle differentiation and maturation, including muscle contraction, sarcomere organization, striated muscle contraction, skeletal muscle contraction, and skeletal muscle fiber development. Correspondingly, enriched CCs included the Z disc, M band, sarcolemma, sarcomere, and troponin complex. MFs reflected this structural specialization, highlighting structural constituents of muscle, actin binding, titin binding, and extracellular matrix interaction. Conversely, downregulated genes in group 1 were enriched in pathways associated with proliferative and biosynthetic activity, such as translation, DNA replication, cell cycle progression, and ribosome biogenesis (Table 1).

**Table 1:** GO enrichment of the down regulated (upper) and up-regulated (lower) genes after 3 days of differentiation. The top 5 biological processes (BP), cellular components (CC), and molecular functions (MF) are represented. In each functional category, terms are ranked according to their adjusted p-values

GO Term	GO ID	Ontology	Genes	Fold Enrichment	Adjusted *p*-value
**GO terms enriched among downregulated genes**					
translation	GO:0006412	BP	83	4.27	8,99E-30
DNA replication	GO:0006260	BP	34	5.22	1,48E-14
DNA repair	GO:0006281	BP	39	3.17	2,86E-08
mitotic cell cycle	GO:0000278	BP	32	3.46	1,04E-07
DNA replication initiation	GO:0006270	BP	11	8.72	1,50E-07
structural constituent of ribosome	GO:0003735	MF	96		2,35E-39
ATP binding	GO:0005524	MF	200		6,29E-09
DNA helicase activity	GO:0003678	MF	14		1,41E-05
microtubule binding	GO:0008017	MF	39		7,59E-05
identical protein binding	GO:0042802	MF	114		7,90E-05
ribosome	GO:0005840	CC	55	4.93	4,42E-23
nucleoplasm	GO:0005654	CC	255	1.65	1,10E-14
nucleolus	GO:0005730	CC	100	2.36	2,57E-14
cytosol	GO:0005829	CC	291	1.54	6,18E-13
kinetochore	GO:0000776	CC	28	5.11	3,01E-12
**GO terms enriched among upregulated genes**					
muscle contraction	GO:0006936	BP	18	5.87	1,21E-07
sarcomere organization	GO:0045214	BP	16	6.05	4,05E-07
striated muscle contraction	GO:0006941	BP	10	7.88	1,36E-05
skeletal muscle contraction	GO:0003009	BP	11	6.5	5,21E-05
skeletal muscle fiber development	GO:0048741	BP	10	6.76	0,000100942
actin binding	GO:0003779	MF	51	2.74	1,21E-08
actin filament binding	GO:0051015	MF	40	3.06	2,64E-08
structural constituent of muscle	GO:0008307	MF	12	6.91	1,76E-06
extracellular matrix structural constituent	GO:0005201	MF	16	3.64	0,000663338
titin binding	GO:0031432	MF	6	8.39	0,001493905
Z disc	GO:0030018	CC	28	5.59	6,54E-13
M band	GO:0031430	CC	11	7.47	2,42E-06
sarcolemma	GO:0042383	CC	16	4.66	9,13E-06
sarcomere	GO:0030017	CC	14	5.09	9,13E-06
troponin complex	GO:0005861	CC	7	10.19	9,13E-06

In group 3 (day 0 vs. day 16), the enrichment of GO terms among upregulated genes was consistent with further maturation of the myotube phenotype, reinforcing the trends observed at earlier stages. Downregulated genes showed significant enrichment in biological processes such as mitotic cell cycle, mRNA splicing, DNA replication, and rRNA processing. Enriched cellular components included the nucleoplasm, nucleolus, cytosol, spliceosome, and centrosome, while molecular functions included RNA binding and ribosomal activity, indicative of reduced proliferative capacity and transcriptional reprogramming during terminal differentiation (Table 2). Volcano plots highlight key differentially expressed genes (DEGs) associated with the enriched GO terms at days 3 and 16 of differentiation (Fig. 6d–e).

**Table 2:** GO enrichment of the down regulated (upper) and up-regulated (lower) genes after 16 days of differentiation. The top 5 biological processes (BP), cellular components (CC), and molecular functions (MF) are represented. In each functional category, terms are ranked according to their adjusted p-values

GO Term	GO ID	Ontology	Genes	Fold Enrichment	Adjusted *p*-value
**GO terms enriched among downregulated genes**					
DNA replication	GO:0006260	BP	35	3.57	1,25E-09
mRNA splicing, via spliceosome	GO:0000398	BP	46	2.93	1,59E-09
rRNA processing	GO:0006364	BP	35	3.16	4,48E-08
DNA repair	GO:0006281	BP	48	2.59	6,45E-08
RNA processing	GO:0006396	BP	39	2.86	8,99E-08
RNA binding	GO:0003723	MF	184	2.35	1,90E-28
ATP binding	GO:0005524	MF	331	1.7	4,17E-22
DNA helicase activity	GO:0003678	MF	20	4.94	4,41E-09
chromatin binding	GO:0003682	MF	74	2.06	1,10E-07
single-stranded DNA binding	GO:0003697	MF	24	3.02	3,88E-05
nucleolus	GO:0005730	CC	171	2.63	2,10E-33
nucleoplasm	GO:0005654	CC	409	1.73	3,27E-30
cytosol	GO:0005829	CC	432	1.48	1,70E-17
U2-type precatalytic spliceosome	GO:0071005	CC	32	4.71	1,15E-14
centrosome	GO:0005813	CC	104	2.15	4,47E-13
**GO terms enriched among upregulated genes**					
sarcomere organization	GO:0045214	BP	19	4.77	2,23E-07
intracellular signal transduction	GO:0035556	BP	96	1.8	4,38E-06
muscle contraction	GO:0006936	BP	18	3.9	4,10E-05
actomyosin structure organization	GO:0031032	BP	14	4.39	9,95E-05
cardiac muscle contraction	GO:0060048	BP	14	4.39	9,95E-05
structural constituent of muscle	GO:0008307	MF	13	4.91	5,35E-05
actin filament binding	GO:0051015	MF	44	2.21	7,63E-05
protein binding	GO:0005515	MF	183	1.4	0,000344618
titin binding	GO:0031432	MF	7	6.42	0,000746079
PDZ domain binding	GO:0030165	MF	21	2.75	0,001263509
Z disc	GO:0030018	CC	33	4.28	8,82E-13
mitochondrion	GO:0005739	CC	154	1.74	2,93E-10
myofibril	GO:0030016	CC	21	4.48	1,30E-08
cytosol	GO:0005829	CC	394	1.32	5,28E-08
cytoplasm	GO:0005737	CC	505	1.27	5,28E-08

To further validate the transcriptional changes observed during myogenic differentiation, the expression of key marker genes was analysed individually. Box plots of *Titin* (*Ttn)*, *Myosin heavy chain 1* (*Myh1)*, *Myosin heavy chain 2* (*Myh2)*, and *Myogenin* (*Myog)* confirmed their progressive upregulation over time (Supplementary Figure S3), with the highest expression levels observed at later stages of differentiation (day 7 and 16). These genes encode structural components or regulators of terminal myogenesis, and their temporal expression patterns are consistent with the formation and maturation of functional myotubes.

### Differential splicing analysis

Analysis of alternative splicing revealed extensive transcriptome remodelling during differentiation. Between day 0 and day 3, 6,718 genes exhibited 23,052 alternative splicing events. Comparisons of day 0 vs. day 16 identified 6,156 genes with 20,524 events, respectively. To interpret the functional impact of these events, GO enrichment analysis was performed, and after redundancy reduction, 4,823 non-redundant terms were found for group 1 and 4,536 for group 3. The treemap visualization highlights the broad functional categories represented at day 3 (Fig. 6f) and day 16 (Fig. 6g). At day 3, enriched terms were dominated by processes related to cell cycle regulation, DNA metabolic processes, catabolic pathways, intracellular signal transduction, and cellular response to stress. At day 16, the enriched BP categories shifted toward functions associated with protein metabolic processes, cellular localization, organelle organization, and multicellular organism development.

The CC-GO (Supplementary Figure S4a-b) and the MF-GO (Supplementary Figure S4c-d) terms suggest that alternative splicing impacts nuclear remodelling together with regulatory and enzymatic functions.

## Discussion

While recent technical advances with 3D engineered muscle tissue cultures have improved the possibilities to model skeletal muscle in vitro (Qazi et al. 2015), these systems have a relatively low throughput, and require technical expertise and specialized equipment. 2D myotube cultures, accessible to virtually any cell culture laboratory and easily adopted to daily use, provide an adequate model for many applications.

Drawbacks of traditional 2D myotube cultures have been poor myotube maturation, and detachment of twitching myotubes from rigid culture surfaces, preventing longer-term cultures. Hydrogel substrates have been developed to provide a mechanical environment that more closely mimics the extracellular matrix (ECM) of native muscle tissue compared to conventional rigid plastic or glass. Muscle cells are highly mechanosensitive, and it is well established that substrate stiffness plays a pivotal role in guiding myogenic differentiation, fusion, and maturation. Differentiation on micropatterned stiff gelatin hydrogels has been shown to provide excellent myotube alignment and maturation (Bettadapur et al. 2016; Denes et al. 2019), but fabrication of micropatterned substrates may be tedious and difficult to scale to small formats and high throughput. The ultra-compliant gelatin hydrogel developed by Jensen and colleagues (2020) allows for good myotube organization and maturation and is easily scalable (Jensen et al. 2020).

The murine C2C12 myoblast cell line remains a widely used in vitro model for skeletal muscle research due to its ability to differentiate into contractile myotubes under appropriate conditions. Despite this, the reproducibility and efficiency of C2C12 differentiation vary widely across laboratories, highlighting the need for optimized, cost-effective, and robust protocols to support consistent myotube formation and maturation. Our study provides a systematic comparison of different differentiation media, supplements, and environmental stimuli aimed at enhancing C2C12 differentiation on compliant hydrogel substrates.

C2C12 myogenic differentiation is classically induced by switching from growth medium containing high serum (typically fetal bovine serum, FBS) to a low-serum differentiation medium, often based on horse serum (HS). However, batch-to-batch variability in HS significantly impacts differentiation efficiency due to inconsistent levels of hormones, growth factors, and cytokines. Biological variation cause serum to vary in composition and to help standardize this commercial media have been developed. Commercial differentiation media have emerged as standardized alternatives to address this issue, though at increased cost. In our laboratory, we observed that supplementing the standard DMEM + 2% HS formulation with 10% Opti-MEM (DMO) consistently improved differentiation outcomes, prompting us to evaluate its performance against PromoCell’s commercial SkMC media system.

Our data show that while the PromoCell SkMC-DM medium supports early differentiation, prolonged exposure leads to deteriorated myotube morphology. In contrast, switching to SkMC-GM or DMO after 5–7 days significantly improved C2C12 differentiation, as evidenced by MyHC expression (Fig. 2). These results suggest that while SkMC-DM provides an initial stimulus, a secondary medium is necessary for sustained myotube formation. Notably, cells differentiated in DMO or switched to DMO maintained thinner, aligned myotubes, while SkMC-GM induced thicker myotubes that appeared less organized. These morphological differences may reflect differential signaling environments that affect fusion and alignment dynamics.

To dissect the contribution of individual components, we examined the effects of insulin and sodium pyruvate supplementation. Insulin is a known myogenic enhancer, promoting myoblast fusion and upregulation of MyHC via PI3K/Akt signaling pathways. Pyruvate, as a metabolic substrate, could theoretically support the bioenergetic demands of differentiation. Indeed, our results confirm that insulin, alone or in combination with pyruvate, increases MyHC, Calsequestrin, Troponin T1 and T3 expression over baseline DMO levels (Fig. 3d-i), with insulin exerting a stronger effect. Notably, supplementation of DM (lacking Opti-MEM) with insulin partially restored differentiation potential, supporting our assumption that insulin is a key factor driving differentiation.

Our findings also highlight the importance of media change frequency. Although daily medium replacement is standard, less frequent changes are often used for convenience or cost-saving. However, reduced frequency negatively impacted differentiation, as seen in both morphological and molecular markers (Fig. 4). While DMO supplemented with insulin and pyruvate (DMO + PI) mitigated this effect to some extent, optimal outcomes were still observed with daily changes, underscoring the role of nutrient turnover and waste removal.

EPS is commonly used to mimic exercise (Lautaoja et al. 2023), but few studies also reported the benefits of EPS in enhancing myotube maturation by mimicking neural input (Fujita et al. 2007; Langelaan et al. 2011). In our current setting of EPS treatment after 3 weeks of differentiation, we found no significant upregulation in total or fiber-type specific myosin isoforms and markers of differentiation following EPS (Fig. 5a–g). However, at 2 weeks of differentiation, EPS treatment revealed more organized sarcomeric α-actinin patterns (Fig. 5h–i).

The goal of our cell culture experiments is to achieve very mature myotubes that resemble skeletal muscle, and we here show data from myotubes that have differentiated into well formed, striated cells that twitch and on protein level show good amount of skeletal muscle markers like myosin and calsequestrin, as well as muscle full length titin (Fig. 1).

Our RNA-seq analysis across four time points further reinforces the extensive transcriptional remodeling that underlies C2C12 differentiation. PCA and sample clustering (Fig. 6a–b) revealed clear temporal segregation, with day 0 and day 16 samples showing maximal divergence. The transition from proliferative myoblasts to mature myotubes was marked by the upregulation of genes involved in sarcomere assembly, contractility, and extracellular matrix organization, and downregulation of genes related to mitosis, DNA replication, and translation (Tables 1 and 2). These results are consistent with known patterns (Tao et al. 2024) of skeletal muscle differentiation and validate our morphological and protein-based findings.

Interestingly, muscle-specific genes such as *Ttn*, *Myh1*, *Myh2*, and *Myog* were significantly upregulated as early as day 3 (Supplementary Fig. S3), suggesting that transcriptional commitment to the myogenic lineage precedes detectable structural differentiation.

Alternative splicing analysis demonstrated that myogenic differentiation is accompanied by extensive transcriptome remodeling, consistent with the dynamic regulation required to transition from proliferative myoblasts to mature myotubes. The high number of splicing events detected between day 0 and day 3 underscores the intense regulatory activity during early lineage commitment. Functional enrichment analysis revealed that early splicing changes predominantly affect genes involved in cell cycle control, DNA metabolism, and stress responses, highlighting the nuclear reprogramming required for differentiation. By contrast, later stages were enriched for processes related to protein metabolism, cellular localization, and organelle organization, indicating that splicing regulation shifts toward maintaining cellular architecture and metabolic activity in mature myotubes. The CC and MF terms further suggest that these splicing events impact both nuclear components and enzymatic/regulatory functions, pointing to a broad influence of alternative splicing on the structural and functional maturation of skeletal muscle cells (Supplementray S4).

## Conclusions

Our study provides a comprehensive assessment of conditions that influence C2C12 differentiation and myotube maturation. We show that DMO—a cost-effective, easily implemented medium—supports robust differentiation, which can be further enhanced by insulin and pyruvate supplementation. Daily media changes remain essential for optimal outcomes, and EPS can contribute to improved sarcomere organization, albeit without large changes in global myosin expression. Our findings not only reinforce key principles of myogenic differentiation but also offer practical guidance for producing mature, functional myotubes for basic and applied muscle research.

## Supplementary Information

Below is the link to the electronic supplementary material.

Supplementary file1 (DOCX 2664 KB)

Supplementary file1 (DOCX 19 KB)

Supplementary file1 (DOCX 15 KB)

Supplementary file1 (DOCX 5102 KB)

## Data Availability

Bam files have been uploaded in NCBI SRA (accession ID: PRJNA1336407).

## Abbreviations

BP: biological process
CASQ1/2: calsequestrin 1/2
CC: cellular component
DM: differentiation medium
DMO: differentiation medium + Opti-MEM
DMO + I: DMO plus insulin-transferrin-selenium
DMO + P: DMO plus pyruvate
DMO + PI: DMO plus insulin-transferrin-selenium and pyruvate
DM + I: DM + insulin-transferrin-selenium
DM + P: DM plus pyruvate
GO: gene ontology
HS: horse serum
I: insulin-transferrin-selenium
MF: molecular function
MyHC: myosin heavy chain
P: pyruvate
TNNT1: Troponin T1
TNNT3: Troponin T3
SkMC-DM: skeletal muscle differentiation medium (PromoCell)
SkMC-GM: skeletal muscle growth medium (PromoCell)
