# Supplementary material for: Optimizing 2D in vitro differentiation conditions for C2C12 murine myoblasts on gelatin hydrogel
Source: J Muscle Res Cell Motil. 2025 Oct 9;46(4):389–405. doi: 10.1007/s10974-025-09711-0 (PMC12717204; doi:10.1007/s10974-025-09711-0)
Supplement: Supplementary file 1 — Supplementary file1 (DOCX 2664 KB) [file 10974_2025_9711_MOESM1_ESM.docx]

**Supplementary Figures**

**Supplementary Figure S1: Promocell media test**

|  | **1 week** | **2 weeks** |
| --- | --- | --- |
| DMO | **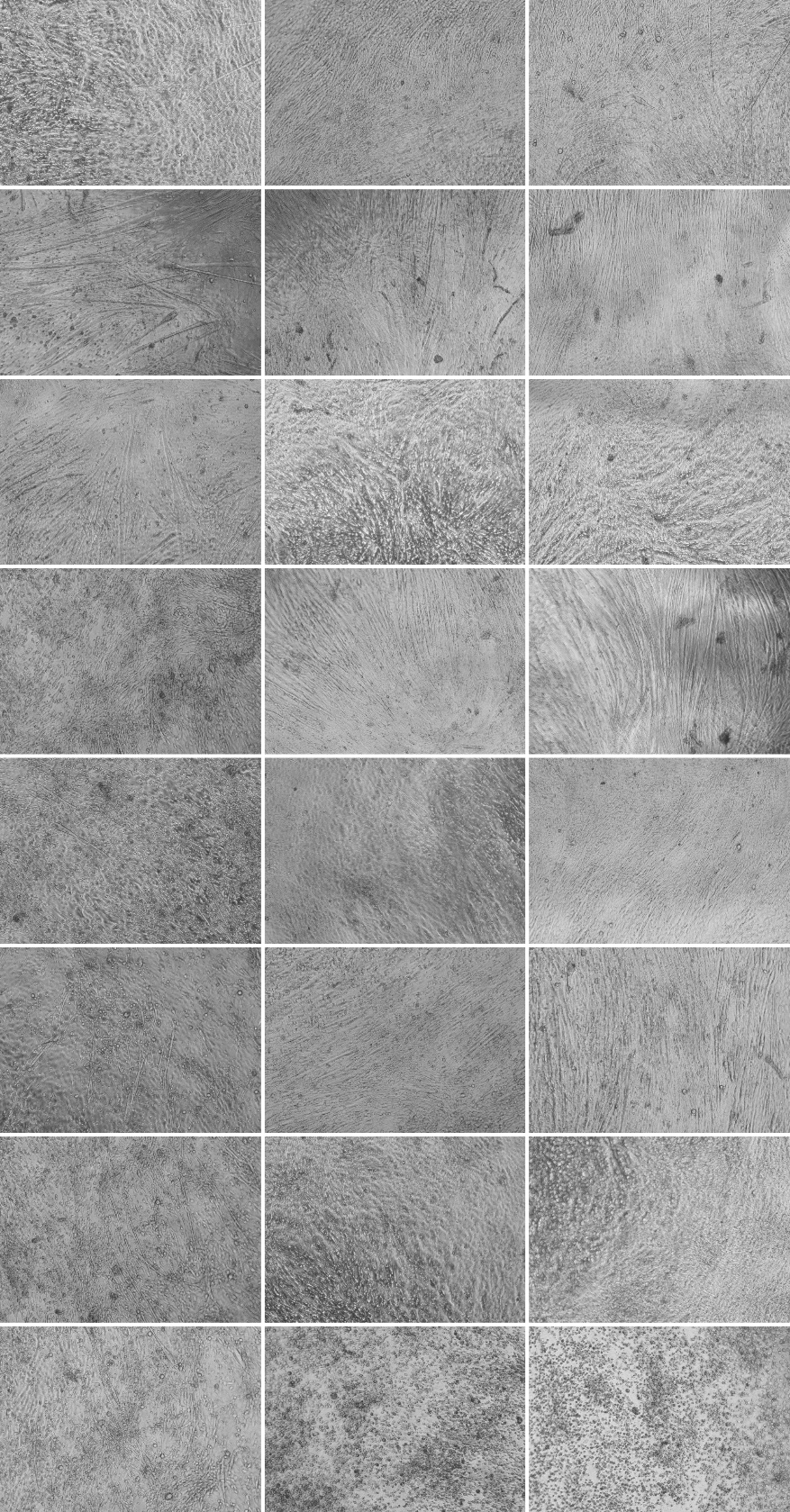** | |
| SkMC-DM -> DMO at d5 |  |  |
| SkMC-DM -> Promo GM at d5 |  |  |
| SkMC-DM -> DMO at d7 |  |  |
| SkMC-DM -> Promo GM at d7 |  |  |
| SkMC-DM -> DMO at d9 |  |  |
| SkMC-DM -> Promo GM at d9 |  |  |
| SkMC-DM |  |  |

Supplementary Figure S1. Promocell media test. Light microscopy of C2C12 cells differentiated on hydrogels for 1 and 2 weeks using DMO and SkMC-DM.

**Supplementary Figure S2**

**
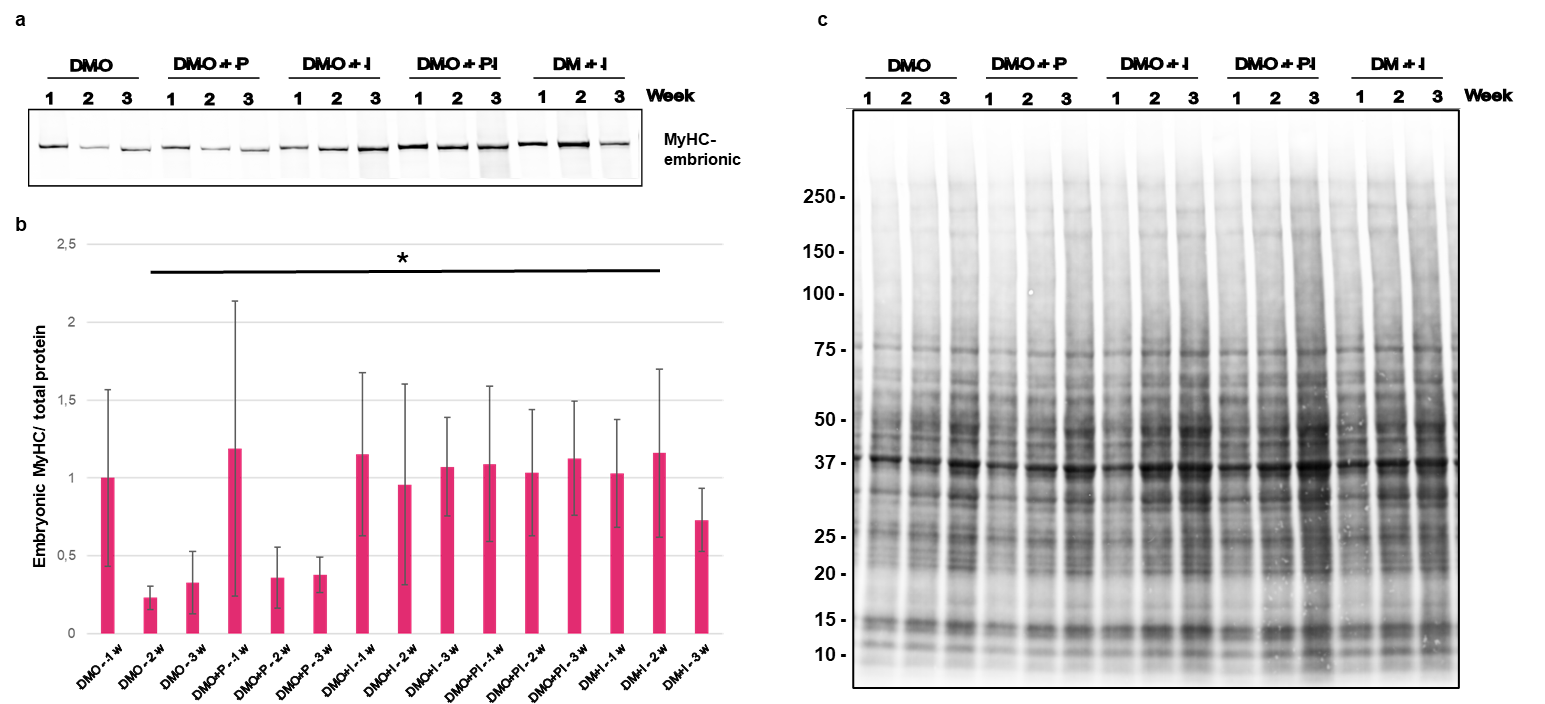
**

Supplementary Figure S2: Effect of media composition to embryonic myosin heavy chain. a) Western blot, b) bar plot represents protein quantification shown as mean±SD of seven wells from three replicate experiments (one asterisk indicates p<0.05), c) total protein stain.

**Supplementary Figure S3**

**
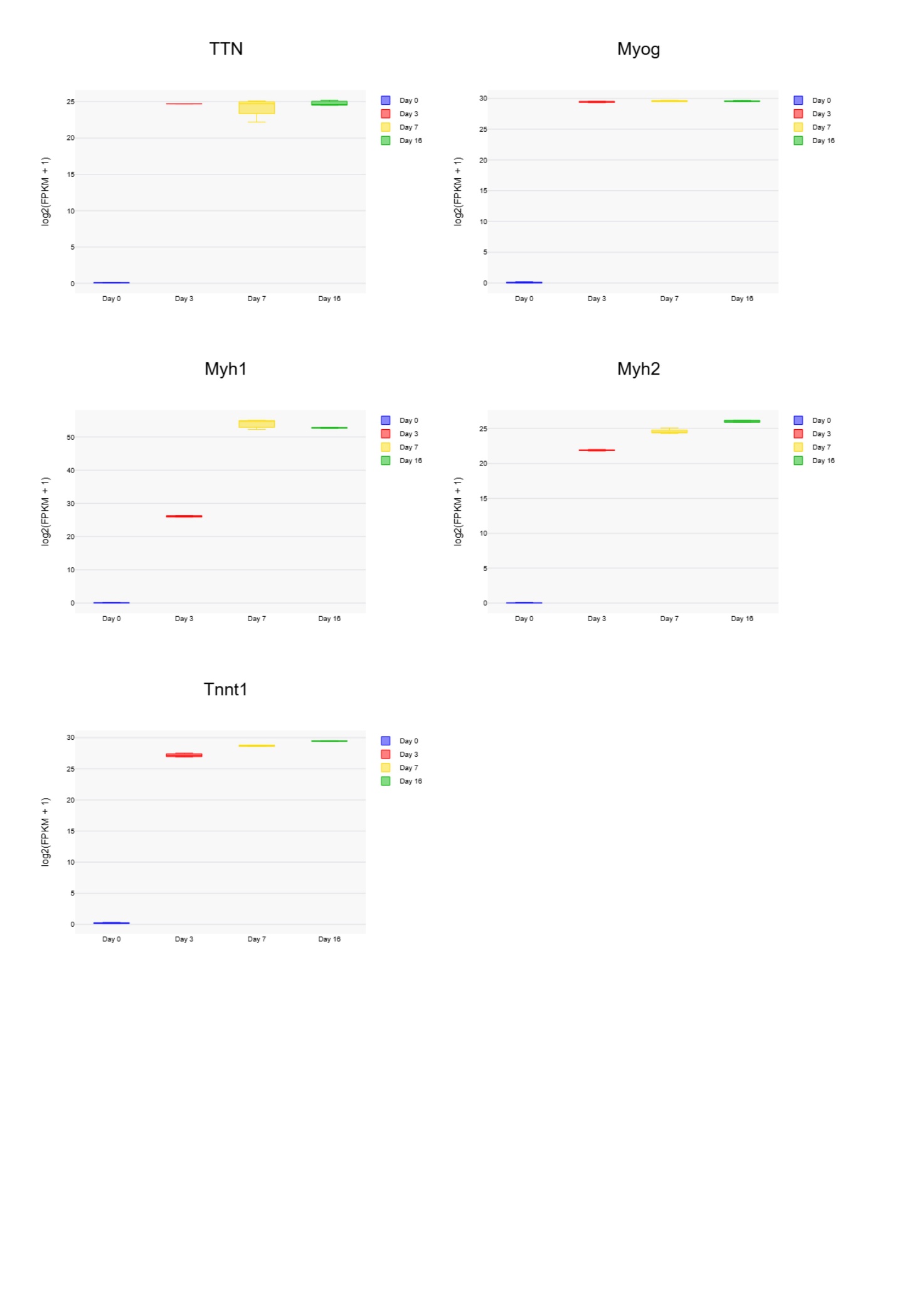
**

Supplementary Figure S3: Box plots of *Titin* (*Ttn)*, *Myosin heavy chain 1* (*Myh1)*, *Myosin heavy chain 2* (*Myh2)*, and *Myogenin* (*Myog)* expression during C2C12 differentiation.

**Supplementary Figure S4**

**
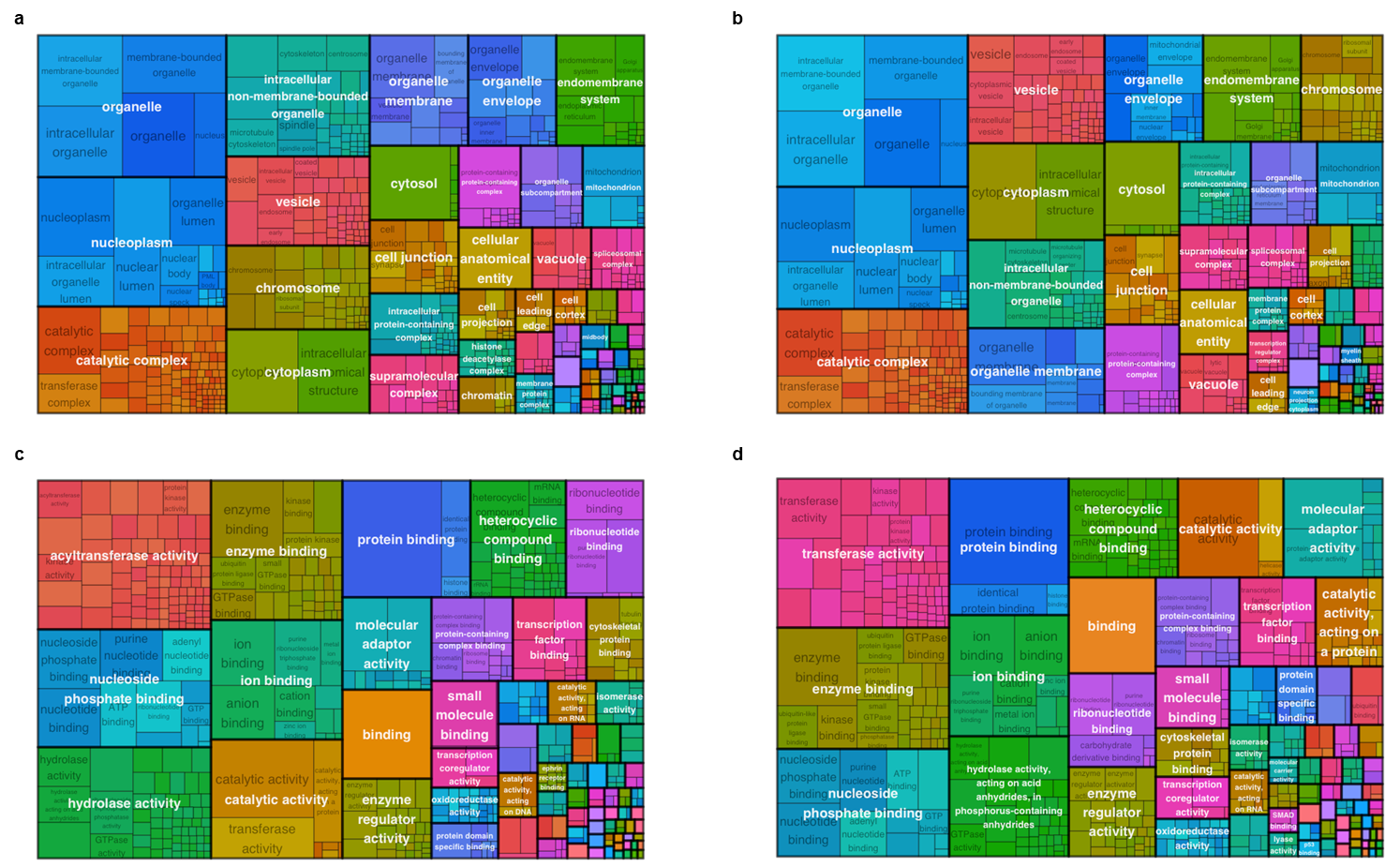
**

Supplementary Figure S4: Treemap charts representing GO terms of differential splicing analysis. a) CC day 3, b) CC day 16, c) MF day 3, d) MF day 16.

**Supplementary Figure S5**

**
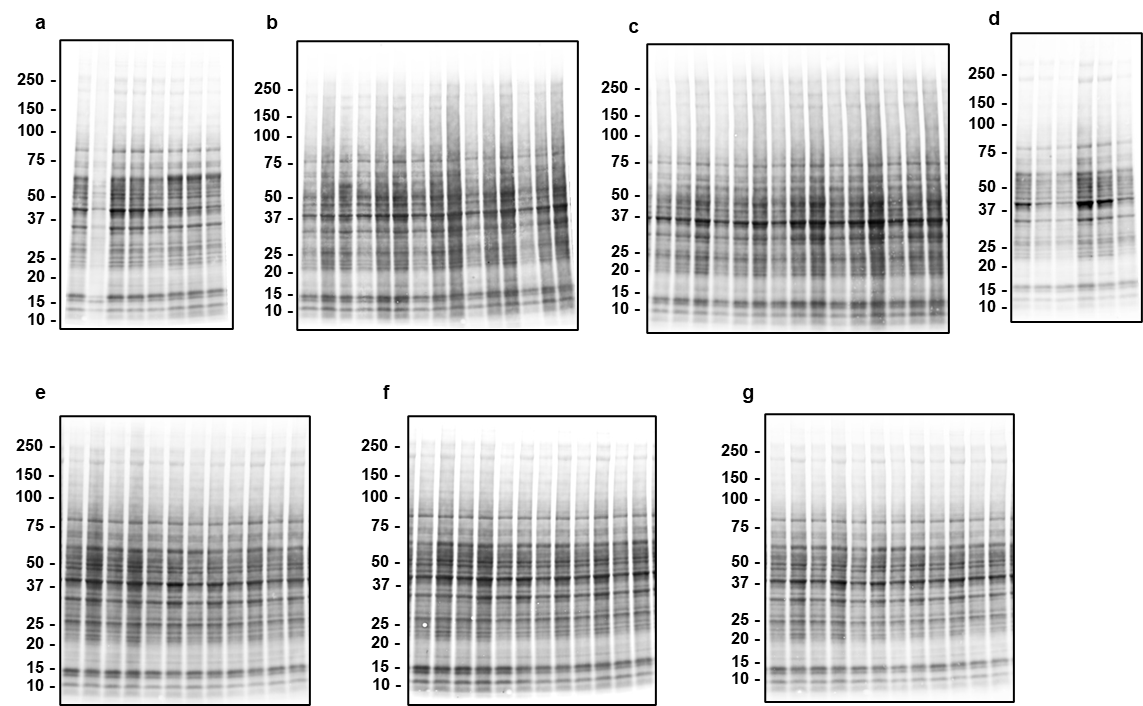
**

Supplementary Figure S5: Total protein stainings for a) MyHC in Figure 2, b) CASQ1/2 in Figure 3, c) TNNT1 and TNNT3 in Figure 3, d) MyHC in Figure 4, e) BA-D5 in Figure 5, f) F59 in Figure 5, g) MF20 and CASQ1/2 in Figure 5.
