# Supplementary material for: Optimizing 2D in vitro differentiation conditions for C2C12 murine myoblasts on gelatin hydrogel
Source: J Muscle Res Cell Motil. 2025 Oct 9;46(4):389–405. doi: 10.1007/s10974-025-09711-0 (PMC12717204; doi:10.1007/s10974-025-09711-0)
Supplement: Supplementary file 2 — Supplementary file1 (DOCX 19 KB) [file 10974_2025_9711_MOESM2_ESM.docx]

**Supplementary Table S1: Composition of media used**

|  | 500 ml DMEM | 5 ml 100x Pen-strep | 5 ml 100x L-glutamine | 10 ml Horse serum | 50 ml Opti-MEM | 5 ml 100x Insulin-Transferrin-Selenium | 5 ml 100x Pyruvate |
| --- | --- | --- | --- | --- | --- | --- | --- |
| DM | x | x | x | x |  |  |  |
| DMO | x | x | x | x | x |  |  |
| DM+I | x | x | x | x |  | x |  |
| DMO+I | x | x | x | x | x | x |  |
| DM+P | x | x | x | x |  |  | x |
| DMO+P | x | x | x | x | x |  | x |
| DM+PI | x | x | x | x |  | x | x |
| DMO+PI | x | x | x | x | x | x | x |

| **Other media used** | |  |
| --- | --- | --- |
| PromoCell GM | Skeletal Muscle Cell Growth Medium | |
| PromoCell DM | Skeletal Muscle Differentiation Medium | |
| GM20 | DMEM + Pen-strep as above, 5 ml 100x GlutaMAX, 100 ml fetal bovine serum | |
