# Supplementary material for: Optimizing 2D in vitro differentiation conditions for C2C12 murine myoblasts on gelatin hydrogel
Source: J Muscle Res Cell Motil. 2025 Oct 9;46(4):389–405. doi: 10.1007/s10974-025-09711-0 (PMC12717204; doi:10.1007/s10974-025-09711-0)
Supplement: Supplementary file 3 — Supplementary file1 (DOCX 15 KB) [file 10974_2025_9711_MOESM3_ESM.docx]

| **Sample Group** | **Samples** | **Time Point** |
| --- | --- | --- |
| **Day 0** | M1–M6 | Undifferentiated myoblasts |
| **Day 3** | M7–M10 | Early Differentiation |
| **Day 7** | M19–M22 | Mid Differentiation |
| **Day 16** | M31–M34 | Mature myotubes |

**Supplementary Table S2: RNAseq samples information**
